# Effect of Synthesis Conditions on Graphene Directly Grown on SiO_2_: Structural Features and Charge Carrier Mobility

**DOI:** 10.3390/nano15171315

**Published:** 2025-08-27

**Authors:** Šarūnas Meškinis, Šarūnas Jankauskas, Lukas Kamarauskas, Andrius Vasiliauskas, Asta Guobienė, Algirdas Lazauskas, Rimantas Gudaitis

**Affiliations:** Institute of Materials Science, Kaunas University of Technology, K. Baršausko 59, LT 51423 Kaunas, Lithuania

**Keywords:** direct synthesis of graphene, FET, PECVD, Raman, self-doping, mobility

## Abstract

Graphene was directly grown on SiO_2_/Si substrates using microwave plasma-enhanced chemical vapor deposition (PECVD) to investigate how synthesis-driven variations in structure and doping influence carrier transport. The effects of synthesis temperature, plasma power, deposition time, gas flow, and pressure on graphene’s structure and electronic properties were systematically studied. Raman spectroscopy revealed non-monotonic changes in layer number, defect density, and doping levels, reflecting the complex interplay between growth, etching, and self-doping mechanisms. The surface morphology and conductivity were assessed by atomic force microscopy (AFM). Charge carrier mobility, extracted from graphene-based field-effect transistors, showed strong correlations with Raman features, including the intensity ratios and positions of the Two-dimension (2D) and G peaks. Importantly, mobility did not correlate with defect density but was linked to reduced self-doping and a weaker graphene–substrate interaction rather than intrinsic structural disorder. These findings suggest that charge transport in PECVD-grown graphene is predominantly limited by interfacial and doping effects. This study offers valuable insights into the synthesis–structure–property relationship, which is crucial for optimizing graphene for electronic and sensing applications.

## 1. Introduction

Graphene is a 2D carbon allotrope with a highly ordered hexagonal architecture [1]. Its remarkable electronic, optical, and mechanical characteristics, including exceptionally high carrier mobility [1], easily adjustable electrical properties [1,2,3,4], and Young’s modulus approaching 1 TPa [5], have established graphene as a highly versatile material for a wide range of electronic and sensing applications [6,7,8,9,10,11,12].

Conventional synthesis approaches, such as mechanical exfoliation and catalytic CVD with metal transfer, can yield high-quality graphene layers but suffer from scalability, contamination, and integration challenges [13,14,15].

A different strategy for graphene fabrication involves growing it directly on dielectric or semiconducting surfaces via plasma-enhanced chemical vapor deposition (PECVD). This method eliminates the need for metal catalysts and avoids the post-growth transfer step, thereby reducing related processing challenges. It also offers enhanced control over graphene morphology and doping, helping to preserve the material’s inherent electronic properties, which is crucial for biosensing and electronic device applications [16,17,18]. Although direct graphene synthesis on dielectric substrates presents clear advantages over transferred graphene, systematic studies explicitly linking synthesis conditions, graphene structure, doping mechanisms, and resulting electronic properties, particularly for graphene-based FET applications, remain limited. Importantly, most prior studies have reported only one charge carrier mobility value for directly synthesized graphene, typically grown using a single set of conditions, without systematically exploring the effects of deposition parameters, graphene structure and self-doping, i.e., unintentional graphene doping arising from interactions with the substrate and/or surface adsorbates [19,20,21,22].

In this study, we investigated the direct synthesis of graphene on SiO_2_ films grown by thermal oxidation using microwave plasma-enhanced chemical vapor deposition (PECVD), with a focus on the relationship between synthesis conditions, graphene structure, and charge transport behavior. Although PECVD is a promising route for scalable, transfer-free graphene growth, factors limiting charge carrier transport in graphene remain insufficiently understood. Directly synthesized graphene is usually nanocrystalline and contains numerous grain boundary defects. These defects are widely believed to limit its electronic performance. However, this assumption remains insufficiently examined in the context of graphene films directly grown on dielectric substrates. Notably, our results demonstrate that substrate-induced self-doping, rather than structural defects, is the dominant factor limiting charge transport in PECVD-grown graphene. These insights offer a deeper understanding of the mobility–defect paradox and highlight the importance of controlling doping and substrate interactions when optimizing graphene for device applications.

## 2. Materials and Methods

Experiments were conducted on silicon substrates coated with a 300 nm SiO_2_ layer, grown by thermal oxidation of single-crystal Si(100) wafers (Sil’tronix, Archamps, France). Graphene was synthesized using the Cyrannus microwave PECVD system (Iplas GmbH, Troisdorf, Germany). More details on the microwave plasma source used are provided in [23,24,25,26]. The microwave plasma frequency was 2.45 GHz. The unit was equipped by the manufacturer with a mechanical pump DUO5M (Pfeiffer, Vantaa, Finland) and an automatic pressure control valve. The base pressure was ~2⋅10^−2^ mBar. Graphene was grown using a gas mixture of methane (CH_4_) and hydrogen (H_2_).

No wet chemical cleaning was performed; substrates were pretreated with hydrogen plasma. Plasma pretreatment was carried out at 1 kW power, 700 °C, 200 sccm H_2_ flow, and 10 mBar pressure for 10 min. After pretreatment, methane flow was initiated, and hydrogen flow was reduced to commence graphene deposition. To prevent direct plasma exposure and unwanted etching, a non-magnetic steel enclosure was employed. All experiments used an H_2_ to CH_4_ flow ratio of 3:1, following our previous research [27,28]. In most cases, gas flows were set to 75 sccm H_2_ and 25 sccm CH_4_. In one test, the gas flow was doubled to 150 sccm H_2_ and 50 sccm CH_4_ to study the effects of precursor supply. The growth temperature ranged from 650 °C to 800 °C, with plasma power set between 0.7 kW and 1.0 kW. Deposition pressure varied from 5 mbar to 30 mbar. Growth durations ranged from 40 to 100 min, with 60 min used most frequently. Synthesis parameters are summarized in Table 1.

To study the electrical properties of the samples, graphene-based field-effect transistors were fabricated. The electrodes consisted of Cr- and Cu-based bilayers deposited on the graphene by thermal evaporation through the mask. Dimensions of each contact were 1.6 mm × 0.4 mm, and the distance between the neighboring contacts was 0.4 mm (Figure S1). A back-gate contact was created by chemically etching the SiO_2_ from the back of the sample to expose the Si surface and depositing an Al film by electron beam evaporation.

The structural properties of the graphene samples were analyzed using a Renishaw inVia Raman spectrometer (Renishaw, Wotton-under-Edge, UK), with excitation provided by a 532 nm laser. Peak deconvolution of the recorded Raman spectra was carried out using XPSPeak 4.1 software. Lorentzian functions were employed to fit the D, G, D’, and 2D bands. The Raman spectra were measured at five points of each sample. Average values of the key Raman spectral parameters are provided in Table S1, while descriptions of these parameters can be found in Table S2.

Atomic force microscopy (AFM) was used for surface morphology and contact current investigations (NanoWizard^®^3, Bruker Nano GmbH, Berlin, Germany). AFM measurements were carried out based on previous research [27]. Notably, an ANSCM-PT silicon tip probe with a Pt/Ir film of 25 ± 5 nm thickness (App-Nano, Mountain View, CA, USA) was used. The tetrahedral tip probe had a spring constant of 1.6 N/m, a radius of curvature (ROC) of 30 nm, a height of 14–16 μm, a scan frequency of 61 kHz, and the applied load was 4 nN. The bias amplitude used for conductivity mapping was ±10 mV. The AFM measurements were carried out in at least three different locations within the sample. A representative image was selected for further analysis.

Electrical properties were studied using a picoammeter/voltage source, a Keithley 6487, to measure the characteristics of the ten FETs in each sample. The average mobility value and standard deviation were calculated to quantify the variability in the electrical properties. This statistical analysis ensured that the reported trends reflect reproducible differences between synthesis conditions rather than random device-to-device variations. The electrodes at the top of graphene were used as source and drain contacts. The common bottom contact was used as a gate electrode.

The direct transconductance method was used to calculate charge carrier mobilities [29]:

(1) $$\mu =g_{m}\frac{L}{WV_{ds}C_{g}}$$

where *g_m_* = *dI_ds_/dV_g_* is the transconductance, *I_ds_* is the drain–source current, *V_g_* is the gate voltage, *L* and *W* are the channel length and width, respectively, *V_ds_* is the voltage between drain and source electrodes, and *C_g_* is the gate capacitance. The gate capacitance was estimated using the equation *C_g_* = *εε*_0_/*t_ox_*. Here, the thermal silicon dioxide dielectric permittivity (*ε*) was 3.9 [30], the oxide layer thickness (*t_ox_*) was 300 nm, and the *ε*_0_ was the vacuum permittivity. The drain–source voltage (*V_ds_*) was set to 0.2 V, as previously suggested [31]. Mobility values were extracted using the direct transconductance method, which inherently includes any contribution from the graphene/metal contact resistance. Since all devices were fabricated with identical geometry and contact metallization, this contribution is expected to be similar across samples, allowing for a reliable comparison of mobility trends. Typical transfer curves used for mobility estimation are presented in Figure S2.

## 3. Results and Discussion

### 3.1. Synthesis Conditions Effects on Graphene Structure

**Figure 1:**
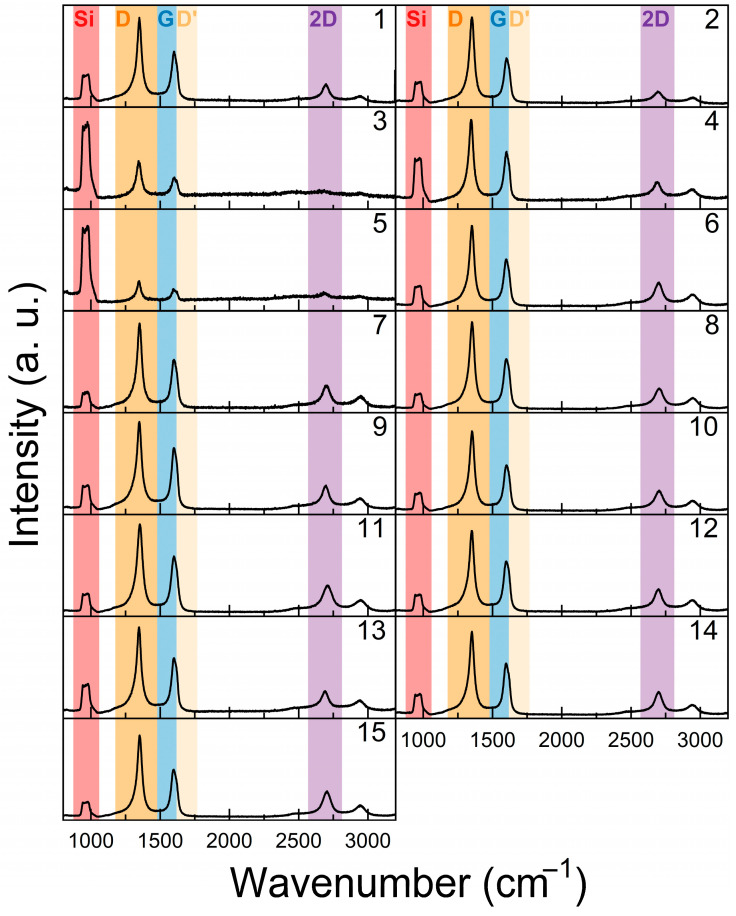
Typical Raman scattering spectra of the graphene. Number in the top right of each Raman spectrum corresponds to sample number.

The Raman scattering spectra of the samples are presented in Figure 1. The G and 2D peaks are typical for graphene. The defect-related D peak is visible. Additionally, the D’ peak, another indicator of graphene defects, was observed as a shoulder of the G peak. A substrate-induced peak found in the 940–990 cm^−1^ range is related to the two-phonon overtones [32]. The most intense substrate-associated peak, attributed to the optical phonon vibrations [32], was observed at ~520 cm^−1^.

**Figure 2:**
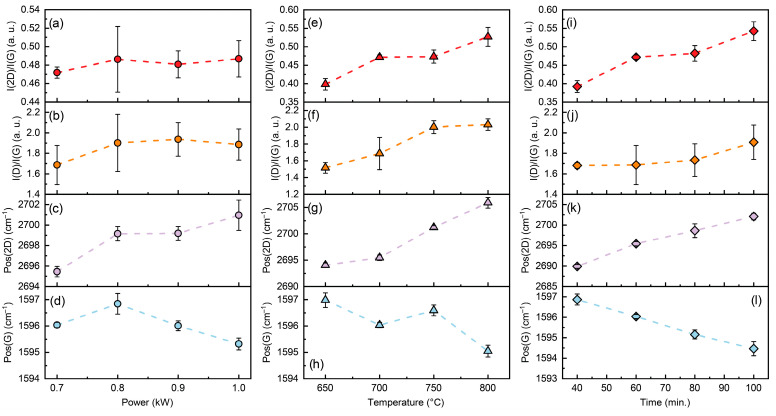
I(2D)/I(G) (red) (**a**,**e**,**i**), I(D)/I(G) (orange) (**b**,**f**,**j**), Pos(2D) (violet) (**c**,**g**,**k**) and Pos(G) (blue) (**d**,**h**,**l**) dependence on graphene synthesis power (circles) (**a**–**d**), temperature (triangles) (**e**–**h**) and time (diamonds) (**i**–**l**). Error bars correspond to Raman parameters dispersion observed within the same graphene specimen. Dashed lines are a guide for the eye.

It is evident that increasing the synthesis temperature and duration reduces the number of graphene layers, as indicated by the rise in the I(2D)/I(G) intensity ratio (Figure 2). The I(D)/I(G) intensity ratio of graphene also increases with extended growth time (Figure 2) and higher methane flow rates (Figure S3). The peak (G) shifts to the lower wavenumber with increased deposition time. Additionally, the tendency of Pos(G) to downshift with increasing synthesis power and temperature is evident. In contrast, Pos(2D) upshifts with graphene growth power, temperature, and time. A non-monotonic dependence of Pos(2D) on synthesis pressure can be seen in Figure S4. A downshift of Pos(2D) was observed at pressures below 10 mBar and above 20 mBar. At the same time, the I(2D)/I(G) ratio decreases, and Pos(G) shifts upward with increasing process pressure (Figure S4).

The ratio of the D to D′ peak intensities (I(D)/I(D′)) shows a non-monotonic dependence on the synthesis parameters (Figure S5). The I(D)/I(D′) values indicate that the main defects are boundary defects or a combination of boundary and on-site defects, typically associated with hydrogen bonding to graphene (see Table S2 and Figure S5). This observation is consistent with the nanocrystalline structure of directly synthesized graphene [32]. Such features are common for graphene grown on semiconducting or dielectric substrates, unless specific substrates, such as hexagonal boron nitride, are chosen or a prolonged, complex two-step growth method is used [32]. The results presented in Figure S5 demonstrate the complex interactions between the dissociation of reactive species, graphene nanocrystal growth, C–H bond incorporation, hydrogen adsorption, and graphene etching during PECVD synthesis.

### 3.2. Raman Spectroscopy and AFM Results

**Figure 3:**
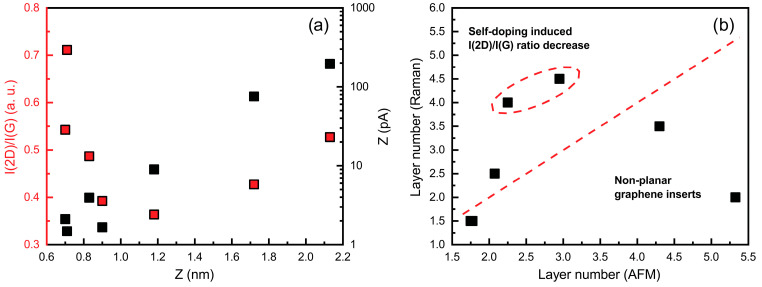
I(2D)/I(G) vs. maximum graphene feature height (red) and maximum current measured by conductivity probe vs. maximum graphene feature height plots (black) (**a**); graphene layer number estimated using I(2D)/I(G) ratio vs. graphene layer number estimated using AFM measurement results (**b**). Dashed lines are visual trends.

**Figure 4:**
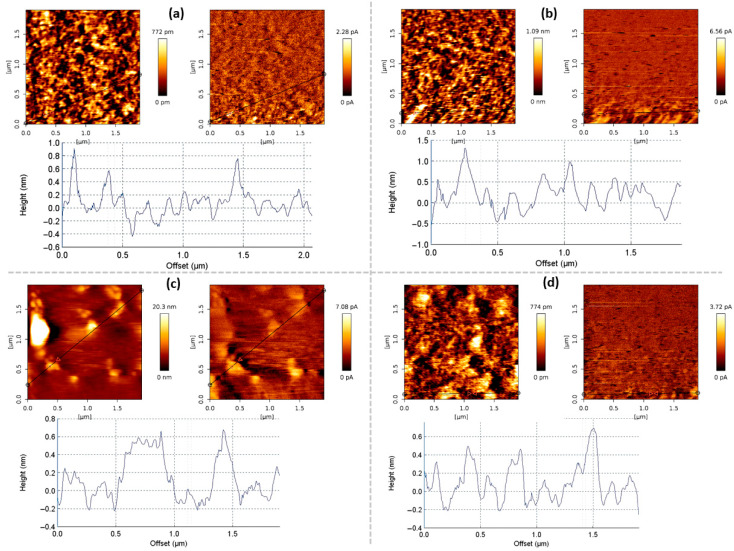
Typical graphene AFM and conductive AFM images. The sample synthesis (plasma power, 0.7 kW) conditions: (**a**) hydrogen flow, 75 sccm; methane flow, 25 sccm; pressure, 10 sccm; synthesis temperature, 700 °C; growth time, 60 min; (**b**) hydrogen flow, 75 sccm; methane flow, 25 sccm; pressure, 10 sccm; synthesis temperature, 650 °C; growth time, 60 min; (**c**) hydrogen flow, 150 sccm; methane flow, 50 sccm; pressure, 10 mBar; temperature, 700 °C; time, 60 min; (**d**) hydrogen flow, 75 sccm; methane flow, 25 sccm; pressure, 10 mBar; temperature, 700 °C; time, 40 min.

Surface topography and local electrical conductivity of selected samples were characterized using conductive atomic force microscopy (C-AFM). AFM analysis of the pristine SiO_2_/Si substrate confirmed its ultra-smooth surface morphology, as shown in Figure S6. To assess the morphology, the maximum height of graphene surface features (Z) was recorded. The apparent thickness of the graphene, which correlates with the number of layers, was approximated both from AFM measurements and evaluation of the intensity ratio of the 2D and G peaks in the Raman spectra, according to the methodologies in [33] and [34], respectively. Figure 3 illustrates that the Raman-based 2D-to-G peak ratio varies non-monotonically with feature height. Initially, the ratio decreases as the feature height increases, suggesting a higher number of graphene layers, as determined by both Raman analysis and surface profiling. However, at higher feature heights, the I(2D)/I(G) ratio shows an upward trend. This can be explained by the presence of non-planar graphene areas similar to those reported in [28]. These non-planar features are clearly visible in the AFM micrographs (Figure 4). The graphene surface conductivity increases markedly with the graphene feature height, indicating its dependence on the presence of non-planar flakes and the number of graphene layers (Figure 3b).

The graphene layer number was estimated using two methods. First, graphene thickness was estimated from AFM topography using a monolayer thickness of 0.41 nm, as reported in [34]. Second, the layer number was determined using the I(2D)/I(G) ratio, as proposed in [33]. While both methods provided generally consistent trends, direct numerical agreement was not always observed. Raman-based estimates sometimes indicated higher layer numbers than AFM-derived values. This may be influenced by factors other than actual thickness. For instance, local variations in doping can significantly modify the I(2D)/I(G) ratio, leading to apparent discrepancies between the two methods [35]. Furthermore, the AFM-derived thickness may be overestimated in the presence of the non-planar graphene features [28], as the tip follows the surface topography rather than the actual layer thickness, reducing the precision of absolute values for ultrathin films. Therefore, AFM and Raman results should be regarded as complementary indicators rather than direct substitutes for determining the layer number.

Overall, the combined AFM and Raman analysis revealed that graphene morphology, layer number, and surface conductivity are closely interrelated, with non-planarity and doping effects contributing to variations in its structural and electronic properties.

### 3.3. Synthesis Conditions Effects on Graphene Electrical Properties

**Figure 5:**
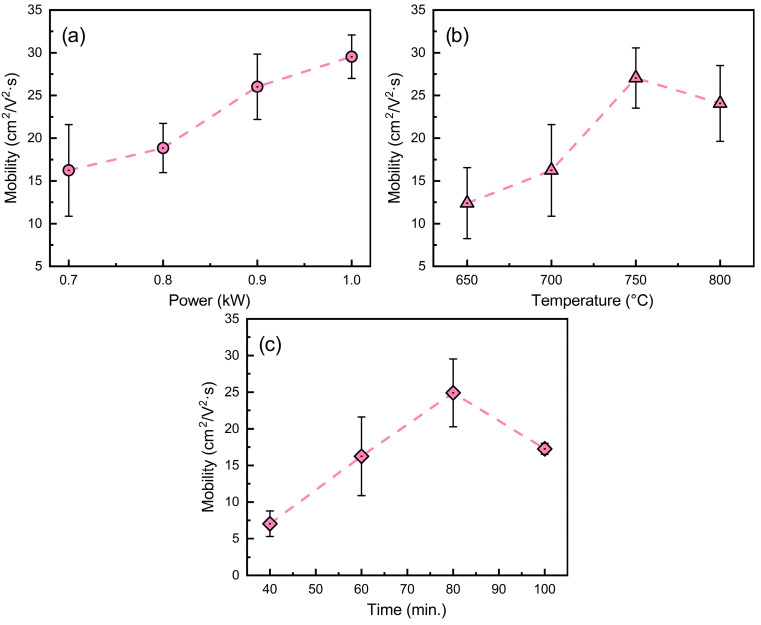
Charge carrier mobility dependence on graphene synthesis power (**a**), temperature (**b**) and time (**c**). Error bars correspond to Raman parameters dispersion observed within the same graphene specimen. Dashed lines are a guide for the eye.

The effects of deposition conditions on graphene mobility were investigated. Mobility was found to generally increase with synthesis power, temperature, and time (Figure 5), although a slight decrease occurred at the highest temperature (800 °C) and longest growth time (100 min). This deviation from the main trend is discussed in Section 3.4. An increase in methane flow from 25 to 50 sccm also promoted the growth of the graphene, resulting in higher charge carrier mobility (Figure S7). The results show that the work pressure must be carefully adjusted to raise mobility (Figure S7). At shorter graphene synthesis times, the charge carrier mobility was substantially lower. These results demonstrate that graphene charge carrier mobility is highly sensitive to deposition conditions, with optimal mobility achieved through careful tuning of synthesis power, temperature, gas flow, pressure, and time.

It is also worth noting that the charge carrier mobility values reported in this research (3–30 cm^2^ V^−1^ s^−1^) are lower than those in some previous studies on directly synthesized graphene, mainly due to the larger FET channel dimensions used here. For example, mobilities of 147 cm^2^ V^−1^ s^−1^ and 707 cm^2^ V^−1^ s^−1^ were reported for directly grown graphene FETs with channel sizes of 20 × 30 µm [21] and 10 µm × 10 µm [20], respectively. Such FET geometry effects have also been observed for transferred graphene grown by CVD on a catalytic foil: a six-fold mobility increase occurred when the channel width was narrowed from 20.7 µm to a few micrometers [36]. It should be noted that the CVD graphene transferred onto the SiO_2_ typically shows higher mobilities, e.g., ~1350 cm^2^ V^−1^ s^−1^ [37], 700–3000 cm^2^ V^−1^ s^−1^ [38], and 1000–6000 cm^2^ V^−1^ s^−1^ [39], although values as low as 89 cm^2^ V^−1^ s^−1^ have also been reported [40]. Larger channels are more susceptible to extrinsic scattering from charged impurities and surface inhomogeneities (see Section 3.4), which degrades the effective mobility [36]. In the present study, we chose large-channel, lithography-free FETs to minimize resist contamination and enable fast, high-throughput fabrication across various synthesis conditions. Reducing channel size in future devices could substantially increase mobility values.

### 3.4. Charge Carrier Mobility in Graphene and Raman Scattering Spectra Parameters

**Figure 6:**
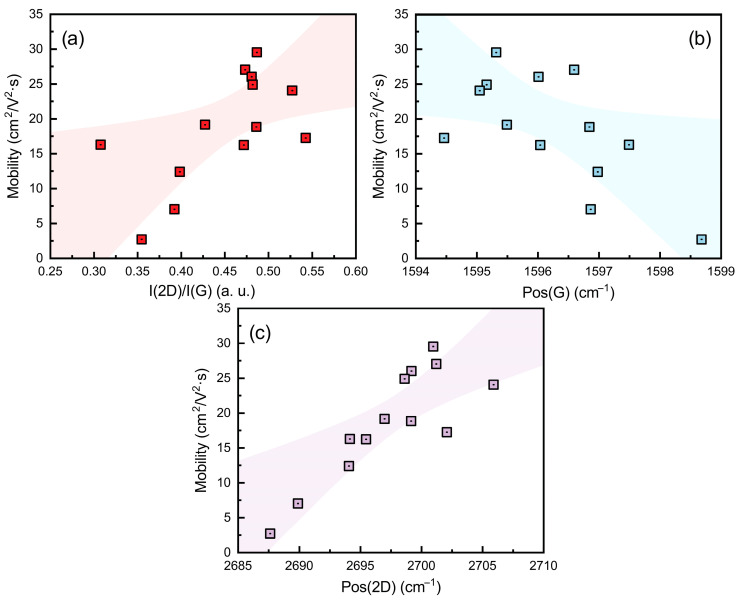
Charge carrier mobility dependence on graphene Raman scattering spectra parameters: I(2D)/I(G) ratio (**a**), Pos(G) (**b**), Pos(2D) (**c**). Shaded area corresponds to a 95% confidence bound. Squares with dot in colors in figures are experimental data.

The relationship between graphene charge carrier mobility and Raman scattering parameters was investigated to elucidate how synthesis conditions influence electronic properties. Additional regression analysis of mobility against defect-sensitive Raman ratios (I(D)/I(G) and I(D)/I(D′)) revealed no significant correlation with defect density or prevailing defect type (Figure S8). This indicates that factors other than structural defects govern mobility behavior. An increase in mobility was associated with a higher I(2D)/I(G) ratio, a Pos(2D) upshift, and a Pos(G) downshift (Figure 6). Additionally, the combination of a Pos(2D) downshift with a Pos(G) upshift (Figure S9) is characteristic of n-type doping in graphene, where higher doping levels promote the Pos(2D) downshift [35,41]. In this work, the n-type doping is attributed to unintentional self-doping arising from interactions with the underlying substrate [27,28]. Such substrate-induced doping occurs when charged impurities at the interface transfer electrons to the graphene, shifting its Fermi level and altering its Raman spectral features [27,28]. These impurities act as long-range Coulomb scattering centers, impairing carrier transport in the two-dimensional lattice and thereby lowering mobility [42]. Overall, the results indicate that substrate-induced self-doping, rather than intrinsic defect scattering, is the primary factor limiting mobility in the directly grown graphene samples.

Beyond charge transfer, shifts in Pos(G) and Pos(2D) also reflect a strain component. The Pos(G) and Pos(2D) values are upshifted compared to typical values for undoped and unstrained graphene (Figure S9), indicating the presence of compressive stress in all graphene samples studied. The slope of the Pos(2D) vs. Pos(G) plot is steeper than expected from pure doping effects (Figure S9), suggesting a combined influence of doping and strain. According to [43], this can be explained by a reduction in compressive stress, combined with increased graphene self-doping. In our samples, such strain differences likely arise from partial detachment of graphene flakes (wrinkles/vertical flakes) due to partial relaxation of the thermal compressive strain [28,44,45] and simultaneous weakening of electrostatic coupling to the substrate [28]. The presence of non-planar graphene in our samples, as revealed by AFM (Figure 3 and Figure 4), supports this interpretation. This strain–doping coupling provides a consistent link between the Raman trends and the mobility changes discussed below [43,46].

The decrease in mobility with increasing graphene layer number observed in the present study initially aligns with prior studies [22,39,47], which attributed this trend primarily to interlayer scattering. However, an increase in graphene’s n-type self-doping with increasing thickness is also observed in our data, as indicated by the Pos(G) downshift and Pos(2D) upshift with the I(2D)/I(G) ratio (Figure S10). Therefore, although interlayer scattering explains part of the mobility trend, additional mechanisms must be involved. The charge transfer from the substrate is strongest for layers closest to the interface and decreases with distance [48]. At the same time, the total capacitance of a multilayer system increases with the number of layers [41]. This larger capacitance corresponds to a higher total density of states [41], enabling the graphene stack to accommodate more transferred charge carriers. In monolayer graphene, the limited density of states near the Dirac point restricts the amount of charge that can be induced [41]. In contrast, in thicker stacks, the injected charge is distributed among more layers, allowing the total doping level to increase, even though the per-layer doping in the uppermost sheets may be lower.

It should be noted that in [37], graphene synthesized via chemical vapor deposition (CVD) and subsequently transferred onto SiO_2_ substrates exhibited decreased mobility with increasing defect density. This was attributed to graphene–SiO_2_ coupling due to defects, which caused a significant rise in charged impurity scattering and a corresponding reduction in mobility [49]. In contrast, directly grown nanocrystalline graphene with a high defect density exhibited substantially higher charge carrier mobility compared to transferred graphene containing few defects [40]. This discrepancy underscores the dominant influence of substrate interactions and doping over defect-related effects.

A decrease in graphene’s mobility with increasing charged impurity density has been reported in numerous studies [50]. As shown in [51], charge carrier mobility in graphene on SiO_2_ is primarily determined by charge-donating impurities from the substrate. Charged impurities located near the graphene layer can severely impair carrier mobility by inducing long-range Coulomb scattering, which disrupts electron and hole transport within the two-dimensional lattice [42]. Therefore, CVD graphene films transferred to substrates with minimal charged impurities exhibit higher carrier mobility [38].

**Figure 7:**
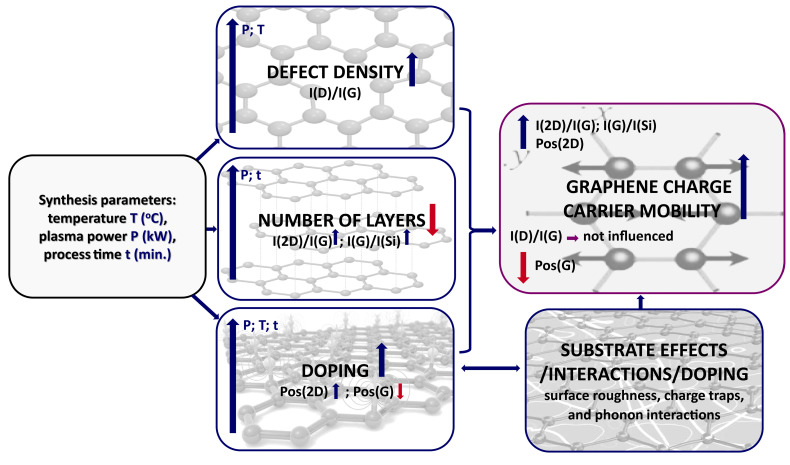
The relationship between the graphene synthesis conditions, graphene structural features and charge carrier mobility in graphene.

Our experimental data also showed that mobility increased with synthesis temperature and time (Figure 5), while the graphene layer number decreased, as indicated by the I(2D)/I(G) analysis (Figure 2 and Figure 6). This trend is attributed to competing mechanisms, where hydrogen etching dominates at extended durations and increased carbon desorption occurs at high temperatures [52]. The synthesis–structure–mobility relationship described above is summarized in Figure 7.

**Figure 8:**
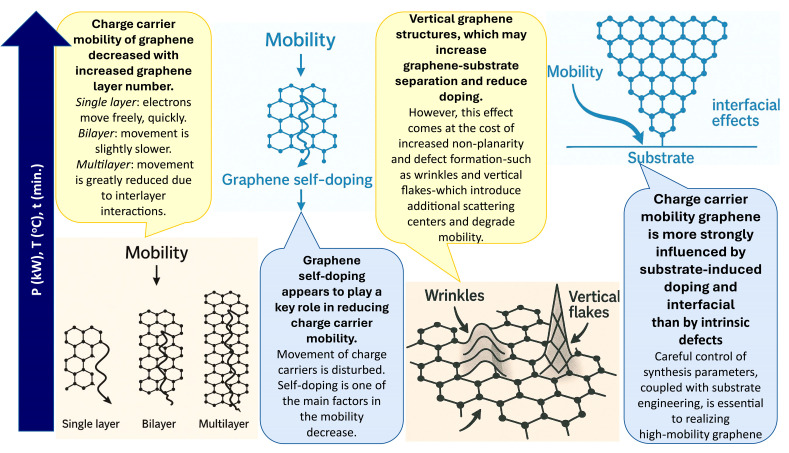
Influence of doping, thickness, defect density or type, and interface effects (substrate engineering) on charge carrier mobility in graphene.

Graphene self-doping reduction with increasing temperature and time may also be linked to thermal stress-induced partial detachment of graphene flakes from the substrate [44,45] (Figure 8). Plasma power-induced self-doping suppression is consistent with previous observations of ion bombardment and electric field effects [53,54]. As reported earlier [28], higher synthesis power, longer growth time, and elevated temperature also promote the formation of the vertical graphene structures, which may increase graphene substrate separation and reduce doping [55]. However, this effect comes at the cost of greater non-planarity and defect formation, such as wrinkles and vertical flakes, which can introduce additional scattering centers and thereby degrade mobility [56].

Pressure effects can be explained by the competition between two processes. Increased pressure reduces the plasma power density per molecule, resulting in a lower dissociation rate and a subsequent decline in the flow of carbon-containing active species and hydrogen atoms [43]. At the same time, it shortens electron mean-free paths and lowers electron temperatures [44]. These factors decrease the gas ionization rate and ion bombardment [44]. Notably, the increase in graphene layer number with process pressure can be explained by the reduced gas ionization [44], resulting in lower ion etching. The non-monotonic behavior of Pos(2D) with pressure likely stems from the interplay of the aforementioned layer number changes and the promotion or suppression of graphene detachment due to altered ion bombardment, as suggested in [44]. Such changes can impact strain and doping levels, shifting Pos(2D) in opposite directions at different pressure ranges and producing the observed trend.

Thus, while instructive, self-doping suppression through partial detachment is not ideal for applications requiring high structural quality. Substrate engineering offers a promising pathway to address this trade-off. Replacing SiO_2_ with hexagonal boron nitride (h-BN) can significantly reduce charged impurity scattering and enhance mobility [57]. Alternatively, modifying SiO_2_ surface polarity—Si-polar surfaces promoting n-type doping and O-polar favoring p-type or neutral doping—can tailor graphene’s behavior [58]. Surface treatments, such as hydrogen plasma exposure, controlled annealing, or chemical passivation, may help minimize doping effects while preserving graphene quality.

Altogether, our findings indicate that in directly synthesized graphene, substrate-induced doping and interfacial effects are the dominant factors influencing charge carrier mobility, whereas intrinsic defects play a secondary role. Careful control of synthesis parameters, together with substrate engineering, is essential to achieve high-mobility graphene for advanced device applications.

## 4. Conclusions

In conclusion, graphene was successfully synthesized directly on thermally oxidized SiO_2_ using microwave PECVD without the need for transfer or catalytic layers. Raman spectroscopy analysis revealed that the graphene structure and doping levels are susceptible to synthesis conditions. The number of graphene layers and defect-related Raman features showed clear correlations with growth time, plasma power, and methane concentration, indicating a balance between carbon supply, etching, and ion bombardment effects. It was found that increased surface roughness correlates with variations in layer number and vertical graphene formation, which in turn affects surface conductivity. Carrier mobility, extracted from graphene FET electrical characteristic measurements, exhibited a strong dependence on Raman parameters such as I(2D)/I(G), Pos(G), and Pos(2D). Despite the presence of grain boundary defects typical of directly grown nanocrystalline graphene, the dominant factor limiting mobility was identified as substrate-induced self-doping, influenced by graphene–SiO_2_ interactions. Increased mobility was observed under conditions that reduced this interaction, likely due to partial delamination or vertical growth. These findings emphasize that optimizing graphene electronic properties via PECVD requires structural control and careful management of substrate-induced doping. Substrate engineering and doping mitigation strategies may further enhance performance in device applications.

## Figures and Tables

**Table 1:** Summary of the parameters used during the synthesis.

No.	T (°C)	P (kW)	H_2_ (sccm)	CH_4_ (sccm)	p (mBar)	t (min)
1	700	0.7	75	25	10	60
2	700	0.7	75	25	20	60
3	700	0.7	75	25	30	60
4	700	0.7	75	25	25	60
5	700	0.7	75	25	5	60
6	700	0.8	75	25	10	60
7	700	0.9	75	25	10	60
8	700	1	75	25	10	60
9	650	0.7	75	25	10	60
10	750	0.7	75	25	10	60
11	800	0.7	75	25	10	60
12	700	0.7	150	50	10	60
13	700	0.7	75	25	10	40
14	700	0.7	75	25	10	80
15	700	0.7	75	25	10	100

## Data Availability

Data are contained within the article and the Supplementary Materials.

## Supplementary Materials

The following supporting information can be downloaded at: https://www.mdpi.com/article/10.3390/nano15171315/s1, Table S1: Graphene Raman scattering spectra parameters (average values); Figure S1: Top view of the sample. D refers to the drain and S refers to the source; Table S2: The dependence of the graphene Raman scattering spectra parameters on the graphene layers number, stress, doping and defects; Figure S2: Typical transfer curves of the graphene-based FETs used for estimation of the mobility. Graphene‘s synthesis conditions were 1—synthesis temperature 700 °C, plasma power 0.7 kW, H_2_ flow 75 sccm, CH_4_ flow 25 sccm, pressure 10 mBar, growth time 60 min; 7—synthesis temperature 700 °C, plasma power 0.9 kW, H_2_ flow 75 sccm, CH_4_ flow 25 sccm, pressure 10 mBar, growth time 60 min; 10—synthesis temperature 750 °C, plasma power 0.7 kW, H_2_ flow 75 sccm, CH_4_ flow 25 sccm, pressure 10 mBar, growth time 60 min; 14—synthesis temperature 700 °C, plasma power 0.7 kW, H_2_ flow 75 sccm, CH_4_ flow 25 sccm, pressure 10 mBar, growth time 80 min; Figure S3: I(D)/I(G) ratio (a), I(2D)/I(G) ratio (b), Pos(G) (c) and Pos(2D) vs. methane gas flow (d); Figure S4: I(2D)/I(G) ratio (a), I(D)/I(G) ratio (b), Pos(G) (c) and Pos(2D) (d) vs. work pressure. Error bars correspond to Raman parameter dispersion observed within the same graphene specimen; Figure S5: I(D)/I(D‘) ratio vs. plasma power (a), deposition time (b), synthesis temperature (c), methane gas flow (d). Error bars correspond to Raman parameter dispersion observed within the same graphene specimen; Figure S6: AFM image of the pristine SiO_2_ surface; Figure S7: Charge carrier mobility vs. work pressure (a) and methane gas flow (b). Error bars correspond to Raman parameter dispersion observed within the same graphene specimen; Figure S8: Mobility vs. I(D)/I(G) (a) and I(D)/I(D’) (b) ratios. Error bars correspond to Raman parameter dispersion observed within the same graphene specimen. The estimated R^2^ values are significantly below the weak dependence threshold (0.25), and the *p*-values are much higher than 0.05 threshold of statistical significance. These results suggest that the presence and type of defects are not the major contributors to mobility; Figure S9: Pos(2D) vs. Pos(G) plots. Shaded area in the bottom picture corresponds to a 95% confidence bound; Figure S10: Pos(G) vs. I(2D)/I(G) (a) and Pos(2D) vs. I(2D)/I(G) (b). Shaded area corresponds to a 95% confidence bound. References [33,43,59,60,61,62,63,64,65,66,67,68,69,70,71,72,73,74] are cited in the supplementary materials.

## References

[1] Du J., Tong B., Yuan S., Dai N., Liu R., Zhang D., Cheng H.M., Ren W. (2022). Advances in flexible optoelectronics based on chemical vapor deposition-grown graphene. Adv. Funct. Mater..

[2] Huang K., Yu X., Cong J., Yang D. (2018). Progress of graphene–silicon heterojunction photovoltaic devices. Adv. Mater. Interfaces.

[3] Li X., Tao L., Chen Z., Fang H., Li X., Wang X., Xu J.-B., Zhu H. (2017). Graphene and related two-dimensional materials: Structure-property relationships for electronics and optoelectronics. Appl. Phys. Rev..

[4] Bonaccorso F., Sun Z., Hasan T., Ferrari A.C. (2010). Graphene photonics and optoelectronics. Nat. Photonics.

[5] Lee C., Wei X., Kysar J.W., Hone J. (2008). Measurement of the elastic properties and intrinsic strength of monolayer graphene. Science.

[6] (2021). Graphene on the pilot line. Nat. Mater..

[7] Colmiais I., Silva V., Borme J., Alpuim P., Mendes P.M. (2022). Towards RF graphene devices: A review. FlatChem.

[8] Liu Z., Geng M., Chen H., Zhang A.-Q., Lu W.B. (2023). A Perspective on the Recent Progress of Graphene in Microwave Applications: Problems, Challenges and Opportunities. IEEE Microw. Mag..

[9] Lukosius M., Lukose R., Lisker M., Luongo G., Elviretti M., Mai A., Wenger C. Graphene Research in 200 mm CMOS Pilot Line. Proceedings of the 2022 45th Jubilee International Convention on Information, Communication and Electronic Technology (MIPRO).

[10] Szunerits S., Rodrigues T., Bagale R., Happy H., Boukherroub R., Knoll W. (2023). Graphene-based field-effect transistors for biosensing: Where is the field heading to?. Anal. Bioanal. Chem..

[11] Wang Z., Yi K., Lin Q., Yang L., Chen X., Chen H., Liu Y., Wei D. (2019). Free radical sensors based on inner-cutting graphene field-effect transistors. Nat. Commun..

[12] Yeh C.-H., Lain Y.-W., Chiu Y.-C., Liao C., Moyano D.R., Hsu S.S.H., Chiu P.-W. (2014). Gigahertz flexible graphene transistors for microwave integrated circuits. ACS Nano.

[13] Liang X., Sperling B.A., Calizo I.G., Cheng G., Hacker C.A., Zhang Q., Obeng Y.S., Yan K., Peng H., Li Q. (2011). Toward clean and crackless transfer of graphene. ACS Nano.

[14] Lupina G., Kitzmann J., Costina I., Lukosius M., Wenger C., Wolff A., Vaziri S., Östling M., Pasternak I., Krajewska A. (2015). Residual metallic contamination of transferred chemical vapor deposited graphene. ACS Nano.

[15] Yi M., Shen Z.-g. (2015). A review on mechanical exfoliation for the scalable production of graphene. J. Mater. Chem..

[16] Li M., Liu D., Wei D., Song X., Wei D., Wee A.T.S. (2016). Controllable Synthesis of Graphene by Plasma-Enhanced Chemical Vapor Deposition and Its Related Applications. Adv. Sci..

[17] Yang C., Bi H., Wan D., Huang F., Xie X., Jiang M. (2013). Direct PECVD growth of vertically erected graphene walls on dielectric substrates as excellent multifunctional electrodes. J. Mater. Chem..

[18] Rehman M.A., Roy S.B., Akhtar I., Bhopal M.F., Choi W., Nazir G., Khan M.F., Kumar S., Eom J., Chun S.-H. (2019). Thickness-dependent efficiency of directly grown graphene based solar cells. Carbon.

[19] Chen J., Wen Y., Guo Y., Wu B., Huang L., Xue Y., Geng D., Wang D., Yu G., Liu Y. (2011). Oxygen-aided synthesis of polycrystalline graphene on silicon dioxide substrates. J. Am. Chem. Soc..

[20] Luo J., Wang J., Xia F., Huang X. (2018). Catalyst-free synthesis of few-layer graphene films on silicon dioxide/Si substrates using ethylene glycol by chemical vapor deposition. Mater. Res. Express.

[21] Du L., Yang L., Hu Z., Zhang J., Huang C., Sun L., Wang L., Wei D., Chen G., Lu W. (2018). Thickness-controlled direct growth of nanographene and nanographite film on non-catalytic substrates. Nanotechnology.

[22] Liu Q., Gong Y., Wang T., Chan W.L., Wu J.Z. (2016). Metal-catalyst-free and controllable growth of high-quality monolayer and AB-stacked bilayer graphene on silicon dioxide. Carbon.

[23] Hopfe V., Spitzl R., Dani I., Maeder G., Roch L., Rogler D., Leupolt B., Schoeneich B. (2005). Remote microwave PECVD for continuous, wide-area coating under atmospheric pressure. Chem. Vap. Depos..

[24] Engemann J., Walter M. (1999). Modelling of microwave plasma sources: Potential and applications. Plasma Phys. Control. Fusion.

[25] Sung-Spitzl H. (2003). Device for the Production of Homogenous Microwave Plasma. U.S. Patent.

[26] Spitzl R., Aschermann B., Walter M. (2001). Microwave Plasma Generator with the Short Cross-Sectional Side of the Resonator Parallel to the Chamber Axis. U.S. Patent.

[27] Meškinis Š., Lazauskas A., Jankauskas Š., Guobienė A., Gudaitis R. (2024). Advancing Graphene Synthesis: Low-Temperature Growth and Hydrogenation Mechanisms Using Plasma-Enhanced Chemical Vapor Deposition. Molecules.

[28] Meškinis Š., Vasiliauskas A., Guobienė A., Talaikis M., Niaura G., Gudaitis R. (2022). The direct growth of planar and vertical graphene on Si(100) via microwave plasma chemical vapor deposition: Synthesis conditions effects. RSC Adv..

[29] Zhong H., Zhang Z., Xu H., Qiu C., Peng L.-M. (2015). Comparison of mobility extraction methods based on field-effect measurements for graphene. AIP Adv..

[30] Wang Y., Yang R., Zhao Y., Li Z., Zhang W., Tian J. (2022). Independently tunable bifunctional terahertz metasurface based on double-layer graphene. Opt. Mater..

[31] Kumar B., Min K., Bashirzadeh M., Farimani A.B., Bae M.-H., Estrada D., Kim Y.D., Yasaei P., Park Y.D., Pop E. (2013). The role of external defects in chemical sensing of graphene field-effect transistors. Nano Lett..

[32] Khan A., Islam S.M., Ahmed S., Kumar R.R., Habib M.R., Huang K.-F., Hu M., Yu X., Yang D. (2018). Direct CVD Growth of Graphene on Technologically Important Dielectric and Semiconducting Substrates. Adv. Sci..

[33] Hwang J.-s., Lin Y.-H., Hwang J.-Y., Chang R., Chattopadhyay S., Chen C.-J., Chen P., Chiang H.-P., Tsai T.-r., Chen L.C. (2013). Imaging layer number and stacking order through formulating Raman fingerprints obtained from hexagonal single crystals of few layer graphene. Nanotechnology.

[34] Yao Y., Ren L., Gao S., Shi L. (2017). Histogram method for reliable thickness measurements of graphene films using atomic force microscopy (AFM). J. Mater. Sci. Technol..

[35] Casiraghi C., Pisana S., Novoselov K.S., Geim A.K., Ferrari A.C. (2007). Raman Fingerprint of Charged Impurities in Graphene. Appl. Phys. Lett..

[36] Lee Y.G., Lim S.K., Kang C.G., Kim Y.J., Choi D.H., Chung H.-J., Choi R., Lee B.H. (2016). Origin of the channel width dependent field effect mobility of graphene field effect transistors. Microelectron. Eng..

[37] Hwang J.-Y., Kuo C.-C., Chen L.C., Chen K.-H. (2010). Correlating defect density with carrier mobility in large-scaled graphene films: Raman spectral signatures for the estimation of defect density. Nanotechnology.

[38] Scarfe S., Cui W.-w., Luican-Mayer A., Ménard J.-M. (2021). Systematic THz study of the substrate effect in limiting the mobility of graphene. Sci. Rep..

[39] Lin Z., Ye X., Han J., Chen Q., Fan P., Zhang H., Xie D., Zhu H., Zhong M. (2015). Precise Control of the Number of Layers of Graphene by Picosecond Laser Thinning. Sci. Rep..

[40] Simionescu O.-G., Avram A., Adiaconiţă B., Preda P., Parvulescu C.C., Năstase F., Chiriac E., Avram M. (2023). Field-Effect Transistors Based on Single-Layer Graphene and Graphene-Derived Materials. Micromachines.

[41] Ji H., Zhao X., Qiao Z., Jung J., Zhu Y., Lu Y., Zhang L.L., MacDonald A.H., Ruoff R.S. (2014). Capacitance of carbon-based electrical double-layer capacitors. Nat. Commun..

[42] Gosling J.H., Makarovsky O., Wang F., Cottam N.D., Greenaway M.T., Patané A., Wildman R.D., Tuck C.J., Turyanska L., Fromhold T.M. (2021). Universal mobility characteristics of graphene originating from charge scattering by ionised impurities. Commun. Phys..

[43] Lee J.E., Ahn G., Shim J., Lee Y.S., Ryu S. (2012). Optical separation of mechanical strain from charge doping in graphene. Nat. Commun..

[44] Bo Z., Yang Y., Chen J., Yu K., Yan J.H., Cen K. (2013). Plasma-enhanced chemical vapor deposition synthesis of vertically oriented graphene nanosheets. Nanoscale.

[45] Soin N., Sinha S., O’Kane C., McLaughlin J.A., Lim T.H., Hetherington C. (2011). Exploring the fundamental effects of deposition time on the microstructure of graphene nanoflakes by Raman scattering and X-ray diffraction. CrystEngComm.

[46] Mamiyev Z., Balayeva N.O., Ghosal C., Zahn D.R., Tegenkamp C. (2025). Confinement induced strain effects in epitaxial graphene. Carbon.

[47] Limbu T.B., Hernández J.C., Mendoza F., Katiyar R.K., Razink J.J., Makarov V.I., Weiner B.R., Morell G. (2018). A Novel Approach to the Layer-Number-Controlled and Grain-Size-Controlled Growth of High Quality Graphene for Nanoelectronics. ACS Appl. Nano Mater..

[48] Peng H., Schröter N.B., Yin J., Wang H., Chung T.F., Yang H., Ekahana S., Liu Z., Jiang J., Yang L. (2017). Substrate doping effect and unusually large angle van Hove singularity evolution in twisted bi-and multilayer graphene. Adv. Mater..

[49] Wu S., Yang R., Cheng M., Yang W., Xie G., Chen P., Shi D., Zhang G. (2014). Defect-enhanced coupling between graphene and SiO_2_ substrate. Appl. Phys. Lett..

[50] Nagashio K., Nishimura T., Kita K., Toriumi A. (2008). Mobility Variations in Mono- and Multi-Layer Graphene Films. Appl. Phys. Express.

[51] Dorgan V.E., Bae M.-H., Pop E. (2010). Mobility and Saturation Velocity in Graphene on SiO_2_. Appl. Phys. Lett..

[52] Chaitoglou S., Bertran E. (2017). Effect of temperature on graphene grown by chemical vapor deposition. J. Mater. Sci..

[53] Santhosh N.M., Filipič G., Tatarova E.S., Baranov O.O., Kondo H., Sekine M., Hori M., Ostrikov K.K., Cvelbar U. (2018). Oriented Carbon Nanostructures by Plasma Processing: Recent Advances and Future Challenges. Micromachines.

[54] Baranov O.O., Levchenko I., Xu S., Lim J.W.M., Cvelbar U., Bazaka K. (2018). Formation of vertically oriented graphenes: What are the key drivers of growth?. 2D Mater..

[55] Kumar K., Kim Y.-S., Yang E.H. (2013). The influence of thermal annealing to remove polymeric residue on the electronic doping and morphological characteristics of graphene. Carbon.

[56] Ni G., Zheng Y., Bae S., Kim H.R., Pachoud A.J., Kim Y.S., Tan C., Im D., Ahn J.-H., Hong B.H. (2012). Quasi-periodic nanoripples in graphene grown by chemical vapor deposition and its impact on charge transport. ACS Nano.

[57] Dean C.R., Young A.F., Meric I., Lee C.H., Wang L., Sorgenfrei S., Watanabe K., Taniguchi T., Kim P., Shepard K.L. (2010). Boron nitride substrates for high-quality graphene electronics. Nat. Nanotechnol..

[58] Kang Y.-J., Kang J., Chang K.J. (2008). Electronic structure of graphene and doping effect on SiO_2_. Phys. Rev. B.

[59] Zhao W., Tan P.H., Liu J., Ferrari A.C. (2011). Intercalation of Few-Layer Graphite Flakes with FeCl3: Raman Determination of Fermi Level, Layer by Layer Decoupling, and Stability. J. Am. Chem. Soc..

[60] Szirmai P., Márkus B.G., Chacón-Torres J.C., Eckerlein P., Edelthalhammer K., Englert J.M., Mundloch U., Hirsch A., Hauke F., Náfrádi B. (2019). Characterizing the maximum number of layers in chemically exfoliated graphene. Sci. Rep..

[61] Childres I., Jauregui L.A., Tian J., Chen Y.P. (2011). Effect of oxygen plasma etching on graphene studied using Raman spectroscopy and electronic transport measurements. New J. Phys..

[62] Sakavičius A., Astromskas G., Bukauskas V., Kamarauskas M., Lukša A., Nargelienė V., Niaura G., Ignatjev I., Treideris M., Šetkus A. (2020). Long distance distortions in the graphene near the edge of planar metal contacts. Thin Solid Films.

[63] Kim S., Ryu S. (2016). Thickness-dependent native strain in graphene membranes visualized by Raman spectroscopy. Carbon.

[64] Armano A., Buscarino G., Cannas M., Gelardi F.M., Giannazzo F., Schilirò E., Agnello S. (2018). Monolayer graphene doping and strain dynamics induced by thermal treatments in controlled atmosphere. Carbon.

[65] Neumann C., Reichardt S., Venezuela P., Drögeler M., Banszerus L., Schmitz M., Watanabe K., Taniguchi T., Mauri F., Beschoten B. (2015). Raman spectroscopy as probe of nanometre-scale strain variations in graphene. Nat. Commun..

[66] Lee U., Han Y., Lee S., Kim J.S., Lee Y.H., Kim U.J., Son H. (2020). Time Evolution Studies on Strain and Doping of Graphene Grown on a Copper Substrate Using Raman Spectroscopy. ACS Nano.

[67] Wu J.-B., Lin M.-L., Cong X., Liu H.-N., Tan P.-H. (2018). Raman spectroscopy of graphene-based materials and its applications in related devices. Chem. Soc. Rev..

[68] Zeng Y., Lo C.-L., Zhang S., Chen Z., Marconnet A. (2020). Dynamically tunable thermal transport in polycrystalline graphene by strain engineering. Carbon.

[69] Mohiuddin T.M.G., Lombardo A., Nair R.R., Bonetti A., Savini G., Jalil R., Bonini N., Basko D.M., Galiotis C., Marzari N. (2009). Uniaxial Strain in Graphene by Raman Spectroscopy: G peak splitting, Gruneisen Parameters and Sample Orientation. Phys. Rev. B..

[70] Ni Z.H., Yu T., Lu Y.H., Wang Y.Y., Feng Y.P., Shen Z.X. (2008). Uniaxial Strain on Graphene: Raman Spectroscopy Study and Band-Gap Opening. ACS Nano.

[71] Chugh S., Mehta R., Lu N., Dios F.D., Kim M.J., Chen Z. (2015). Comparison of graphene growth on arbitrary non-catalytic substrates using low-temperature PECVD. Carbon.

[72] Eckmann A., Felten A., Mishchenko A., Britnell L., Krupke R., Novoselov K.S., Casiraghi C. (2012). Probing the Nature of Defects in Graphene by Raman Spectroscopy. Nano Lett..

[73] Pereira L.M., Sanchez Rodrigues V., Freires F.G.M. (2024). Use of Partial Least Squares Structural Equation Modeling (PLS-SEM) to Improve Plastic Waste Management. Appl. Sci..

[74] Di Leo G., Sardanelli F. (2020). Statistical significance: P value, 0.05 threshold, and applications to radiomics—reasons for a conservative approach. Eur. Radiol. Exp..

