# Supplementary material for: Effect of Synthesis Conditions on Graphene Directly Grown on SiO2: Structural Features and Charge Carrier Mobility
Source: Nanomaterials (Basel). 2025 Aug 27;15(17):1315. doi: 10.3390/nano15171315 (PMC12429864; doi:10.3390/nano15171315)
Supplement: Supplementary file 1 [file nanomaterials-15-01315-s001.zip › nanomaterials-3780110-supplementary.pdf]

Supporting Information

Article

Effect of Synthesis Conditions on Graphene Directly Grown on SiO_2: Structural Features and Charge Carrier Mobility

Šarūnas Meškinis *, Šarūnas Jankauskas, Lukas Kamarauskas, Andrius Vasiliauskas, Asta Guobienė, Algirdas Lazauskas * and Rimantas Gudaitis

Institute of Materials Science, Kaunas University of Technology, K. Baršausko 59, LT 51423 Kaunas, Lithuania; sarunas.jankauskas@ktu.lt (Š.J.); sarunas6@gmail.com (L.K.); andrius.vasiliauskas@ktu.lt (A.V.); asta.guobiene@ktu.lt (A.G.); rimantas.gudaitis@ktu.lt (R.G.)

* Correspondence: sarunas.meskinis@ktu.lt (Š.M.); algirdas.lazauskas@ktu.edu (A.L.); Tel.: +370-61554257 (Š.M.); +370-67173375 (A.L.)

Table S1. Graphene Raman scattering spectra parameters (average values).

No.	Pos(D) (cm^{-1})	Pos(G) (cm^{-1})	Pos(2D) (cm^{-1})	I(2D)/I(G)	I(D)/I(G)	I(D)/I(D')
1	1348	1596	2695	0.47	1.69	2.98
2	1349	1597	2694	0.31	2.21	4.31
3	1345	1598	2677	0.29	1.95	3.57
4	1346	1599	2688	0.35	1.92	3.94
5	1346	1595	2686	0.71	2.12	3.17
6	1351	1597	2699	0.49	1.90	3.64
7	1350	1596	2699	0.48	1.94	3.65
8	1350	1595	2701	0.49	1.89	3.27
9	1348	1597	2694	0.40	1.52	2.95
10	1351	1597	2701	0.47	2	3.47
11	1353	1595	2706	0.53	2.03	3.16
12	1350	1595	2697	0.43	2.03	3.41
13	1346	1597	2690	0.39	1.68	3.33
14	1350	1595	2699	0.48	1.73	3.22
15	1351	1594	2702	0.54	1.91	3.64

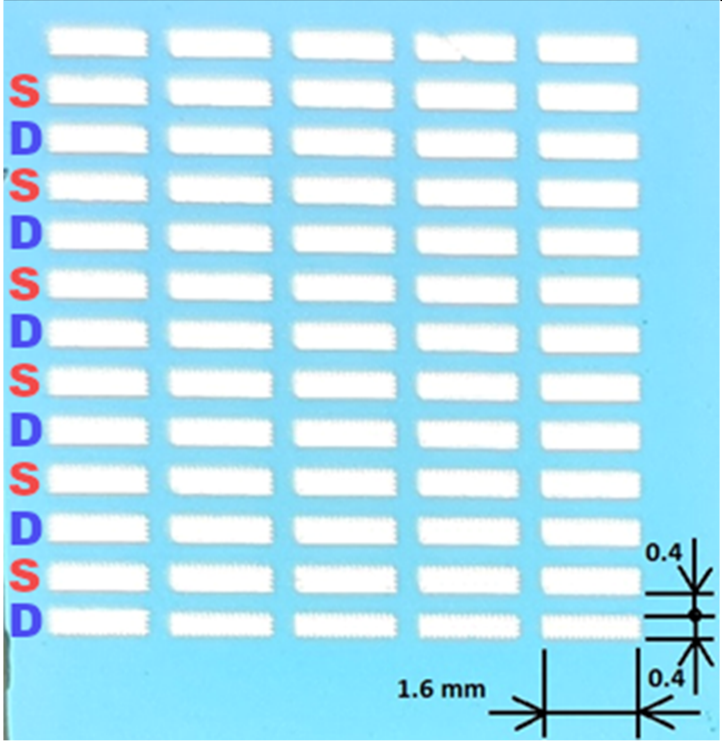

Figure S1. Top view of the sample. D refers to the drain and S refers to the source.

Table S2. The dependence of the graphene Raman scattering spectra parameters on the graphene layer number, stress, doping and defects.

Parameter of the graphene Raman scattering spectra	Graphene layers number (n)	Stress	Doping (p-type)	Doping (n-type)	Defects
I(2D)/I(G)	Decrease with layer number by a law $0.63-0.0595\cdot n$ (for n>1) [33]	N.d.	Decrease with doping [59].	Decrease with doping [60].	Decrease with defects density [61]
I(D)/I(G)	N.d.	N.d.	N.d.	N.d.	Increase with defects density up to ratio value 4 and then decrease [61]
Pos(G)	Downshifts with increased layer number [33]	Shifts to the higher wavenumbers with compressive stress [43, 62-66]<br>Shifts to the lower wavenumbers with tensile stress [67-71]	Shifts to the higher wavenumbers with increased acceptor density [62-65, 67]	At the first no clear shift, afterward, shifts to the lower wavenumbers with increased dopant density [43, 67]	N.d.
Pos(2D)	Upshifts with increased layer number [33]	Shifts to the higher wavenumbers with compressive stress [43, 62-66]<br>Shifts to the lower wavenumbers with tensile stress [67-70]	Shifts to the higher wavenumbers with increased dopant density [62-67]	Shifts to the lower wavenumbers with increased dopant density[43, 67]	N.d.
I(D)/I(D′)	N.d.	N.d.	N.d.	N.d.	1.3 - the on-site defects;<br>3.5 - boundary-like defects;<br>7 - vacancy-like defects;<br>10.5 - hopping defects;<br>13 - sp^3 related defects [72]

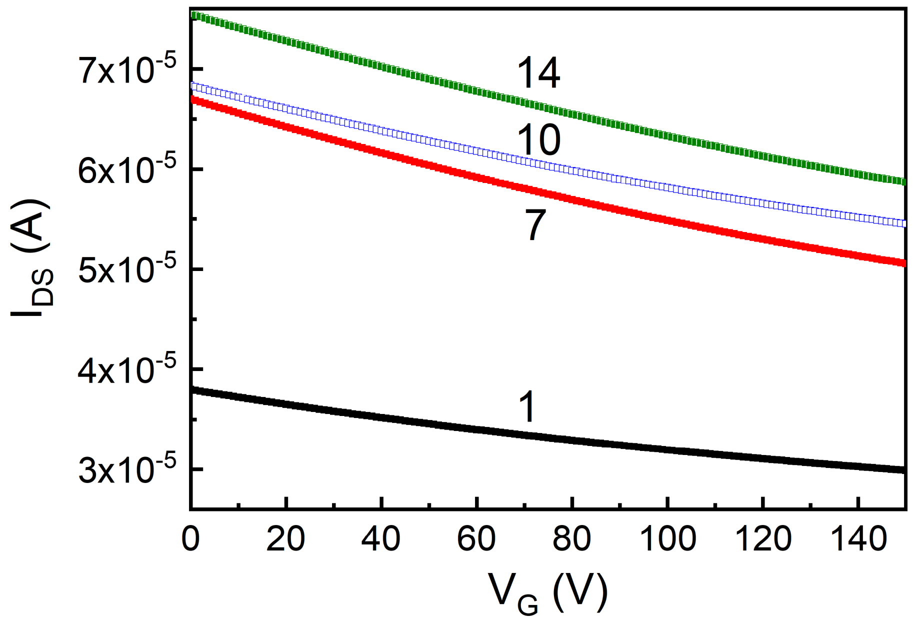

Figure S2. Typical transfer curves of the graphene-based FETs used for estimation of the mobility. Graphene's synthesis conditions were 1 – synthesis temperature 700°C, plasma power 0.7 kW, H_2 flow 75 sccm, CH_4 flow 25 sccm, pressure 10 mBar, growth time 60 min; 7 - synthesis temperature 700°C, plasma power 0.9 kW, H_2 flow 75 sccm, CH_4 flow 25 sccm, pressure 10 mBar, growth time 60 min; 10 - synthesis temperature 750°C, plasma power 0.7 kW, H_2 flow 75 sccm, CH_4 flow 25 sccm, pressure 10 mBar, growth time 60 min;; 14 - synthesis temperature 700°C, plasma power 0.7 kW, H_2 flow 75 sccm, CH_4 flow 25 sccm, pressure 10 mBar, growth time 80 min.

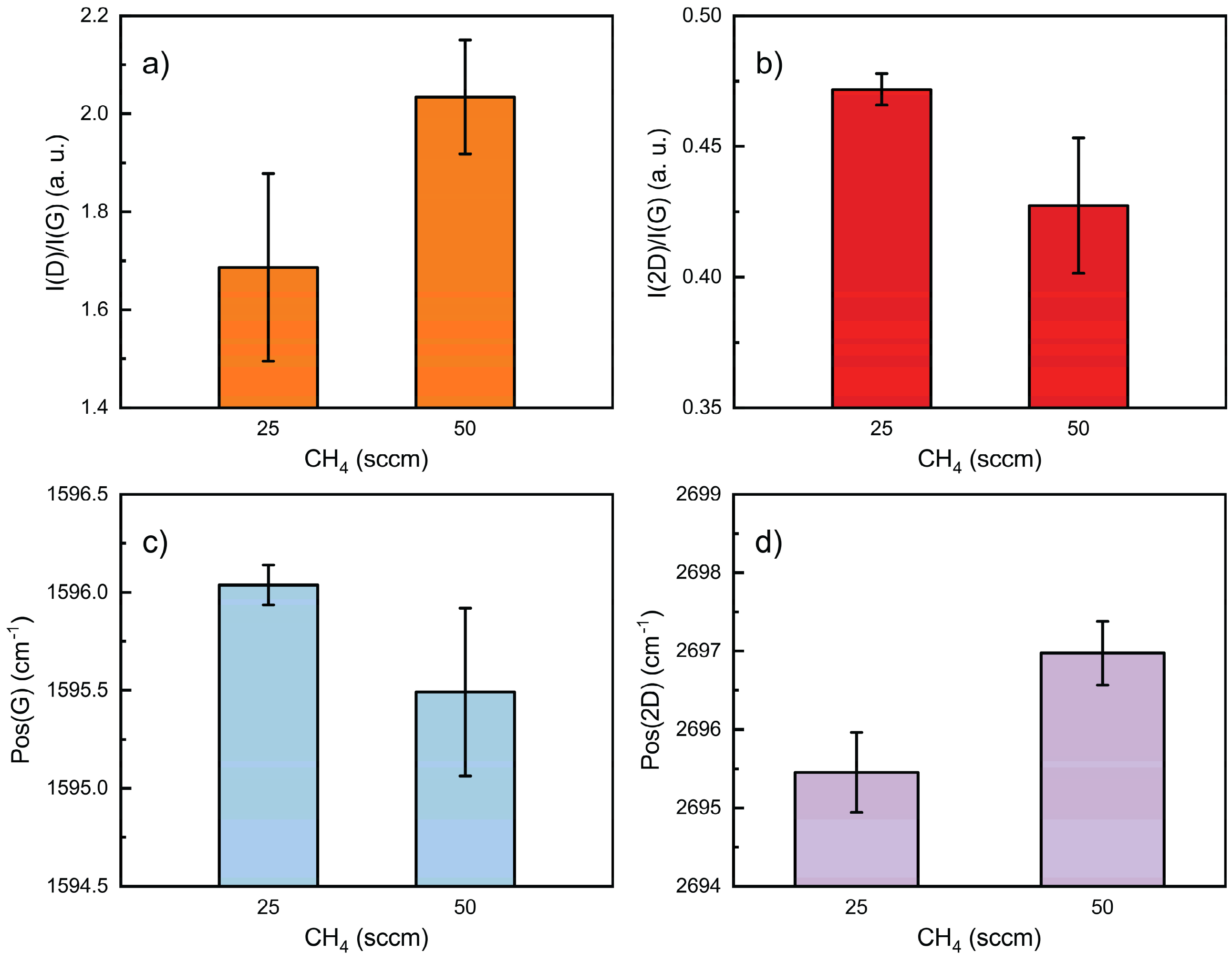

Figure S3. I(D)/I(G) ratio (a), I(2D)/I(G) ratio (b), Pos(G) (c) and Pos(2D) vs. methane gas flow (d).

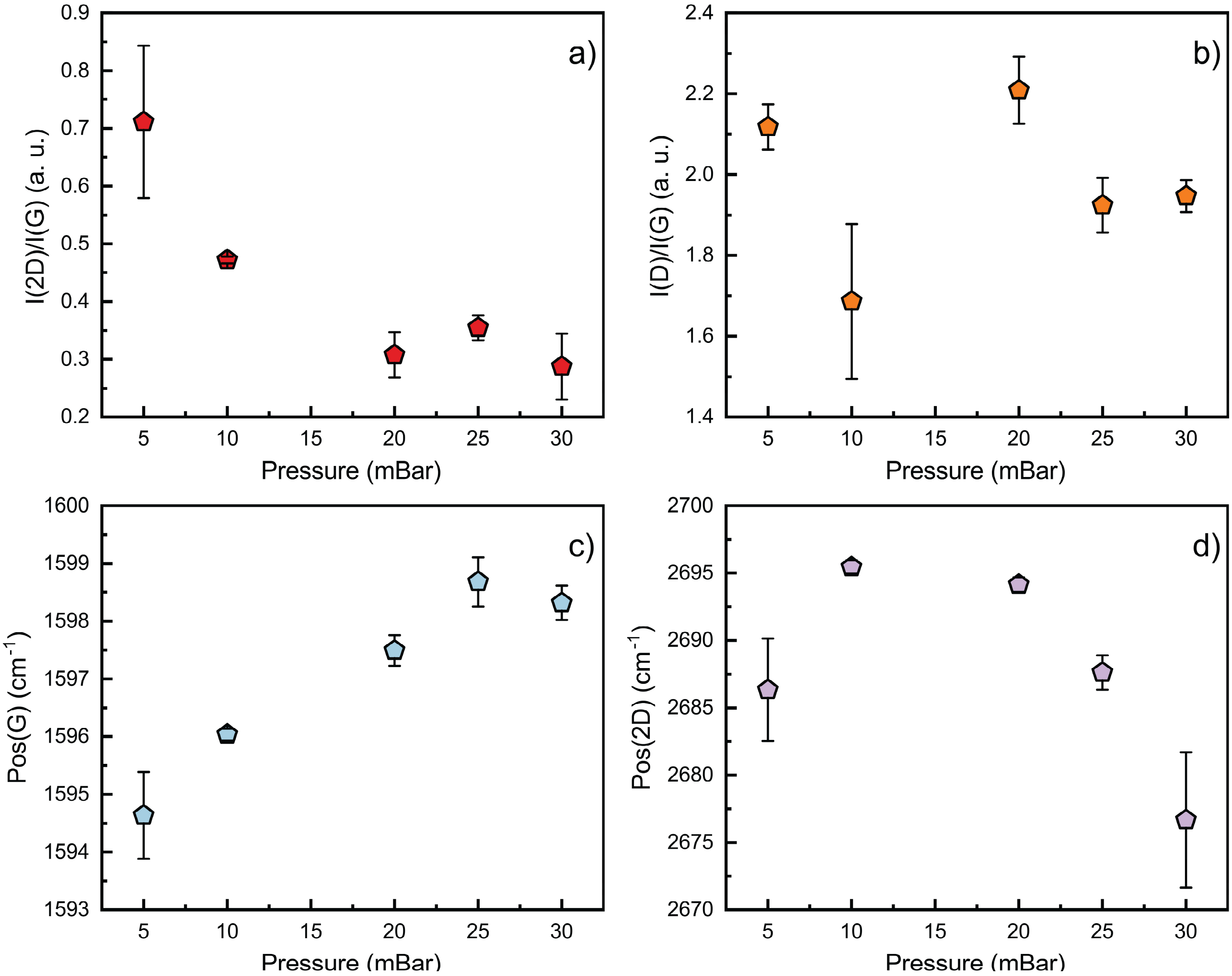

Figure S4. I(2D)/I(G) ratio (a), I(D)/I(G) ratio (b), Pos(G) (c) and Pos(2D) (d) vs. work pressure. Error bars correspond to Raman parameter dispersion observed within the same graphene specimen.

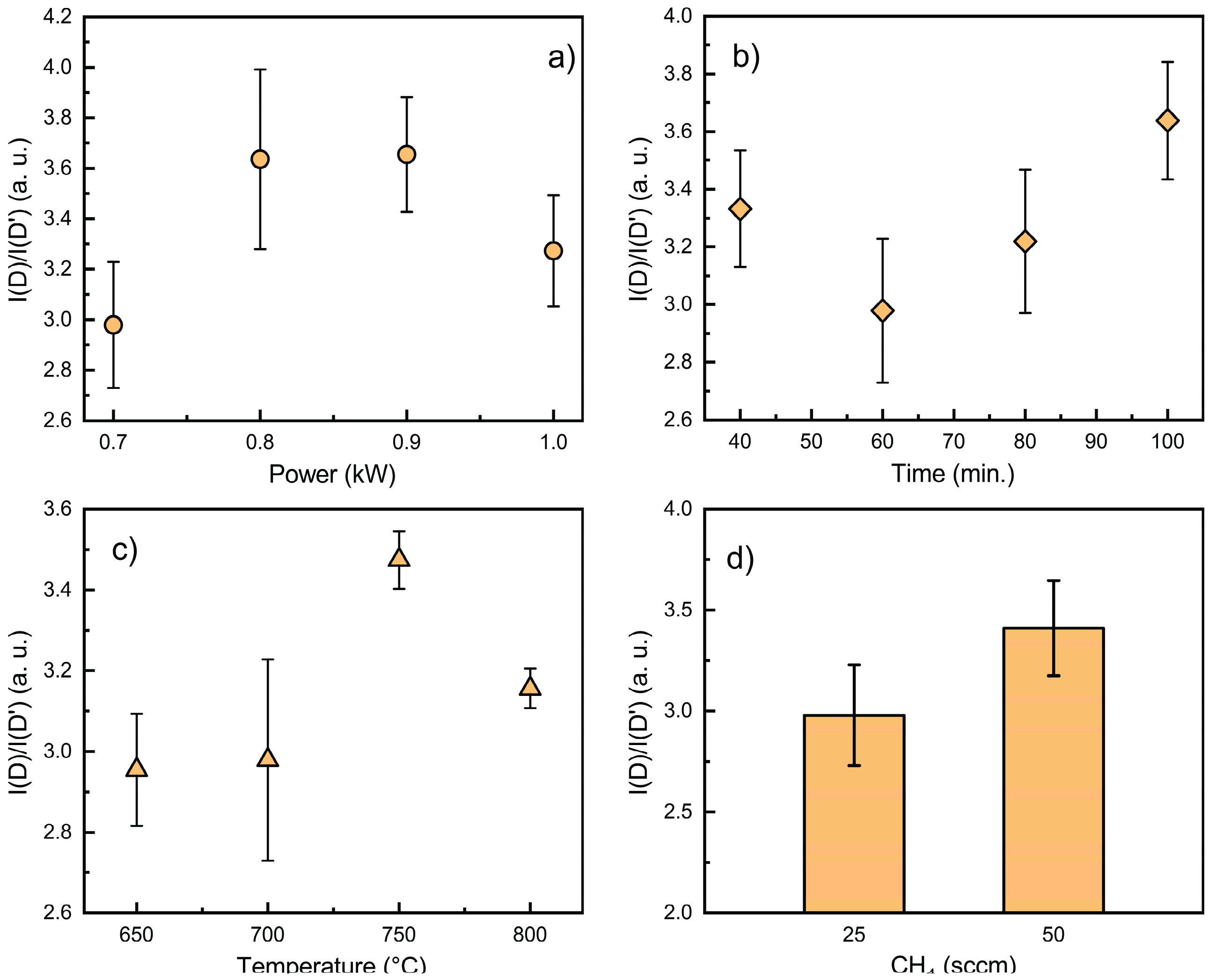

Figure S5. I(D)/I(D′) ratio vs. plasma power (a), deposition time (b), synthesis temperature (c), methane gas flow (d). Error bars correspond to Raman parameter dispersion observed within the same graphene specimen.

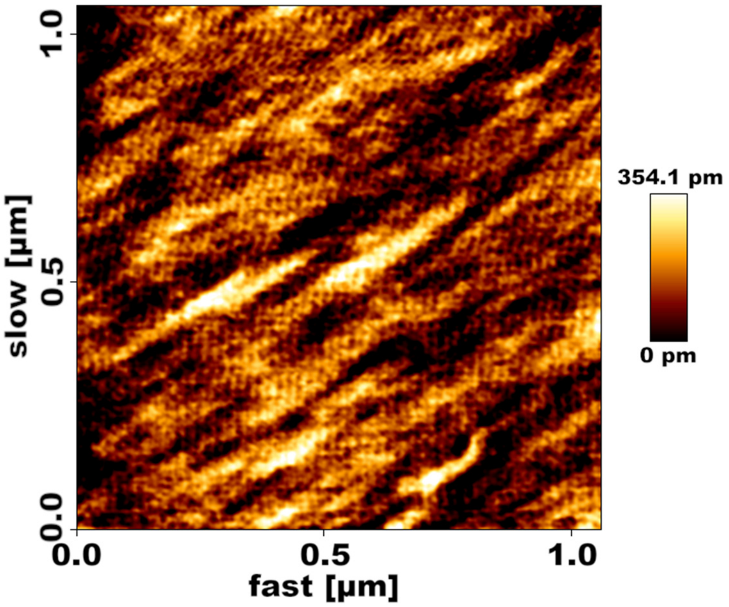

Figure S6. AFM image of the pristine SiO_2 surface.

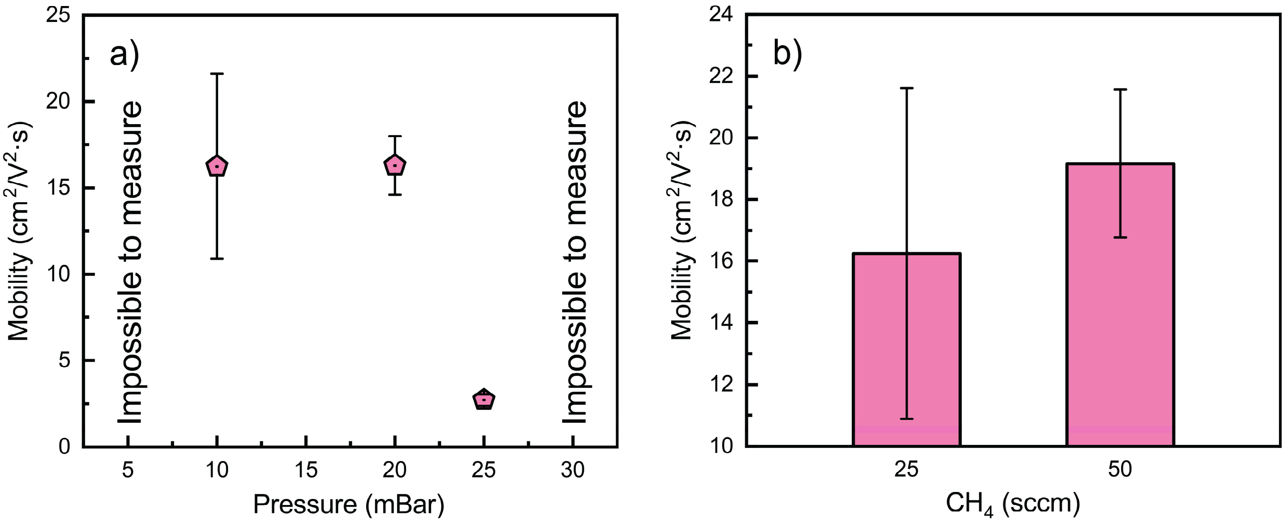

Figure S7. Charge carrier mobility vs. work pressure (a) and methane gas flow (b). Error bars correspond to Raman parameter dispersion observed within the same graphene specimen.

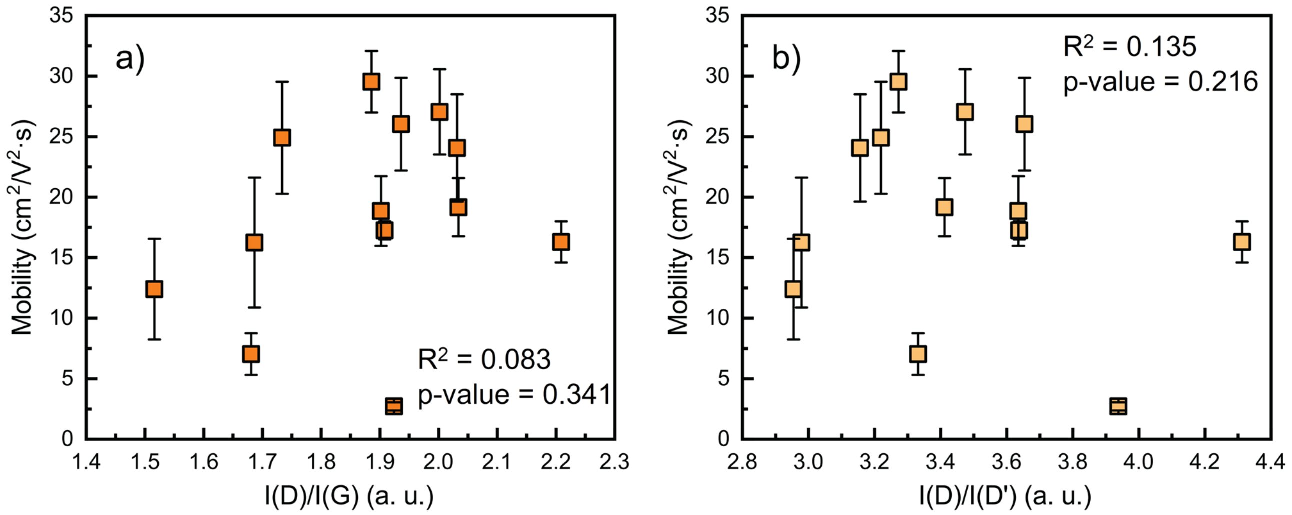

Figure S8. Mobility vs. I(D)/I(G) (a) and I(D)/I(D′) (b) ratios. Error bars correspond to Raman parameter dispersion observed within the same graphene specimen. The estimated R^2 values are significantly below the weak dependence threshold (0.25) [73], and the p-values are much higher than 0.05 threshold of statistical significance [74]. These results suggest that the presence and type of defects are not the major contributors to mobility.

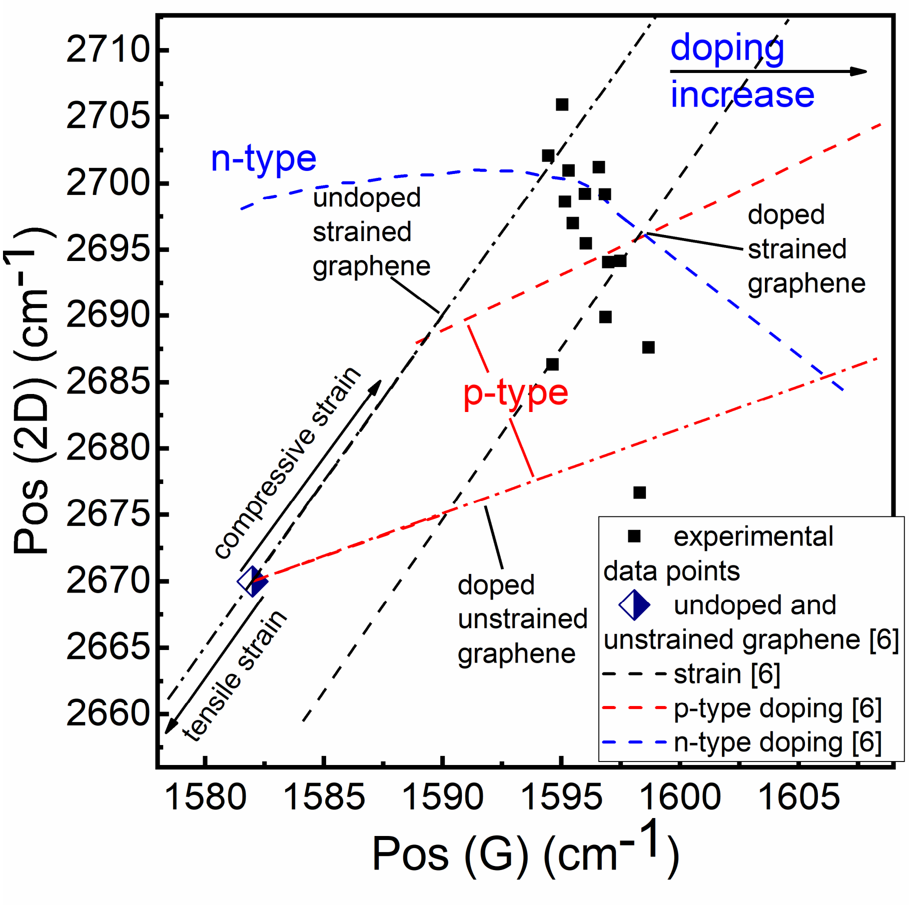

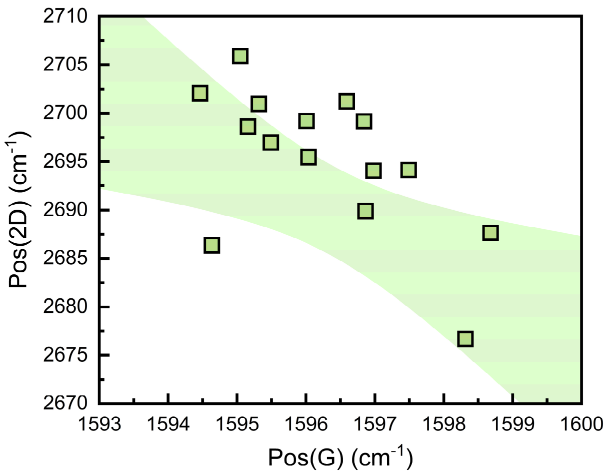

Figure S9. Pos(2D) vs. Pos(G) plots. Shaded area in the bottom picture corresponds to a 95% confidence bound.

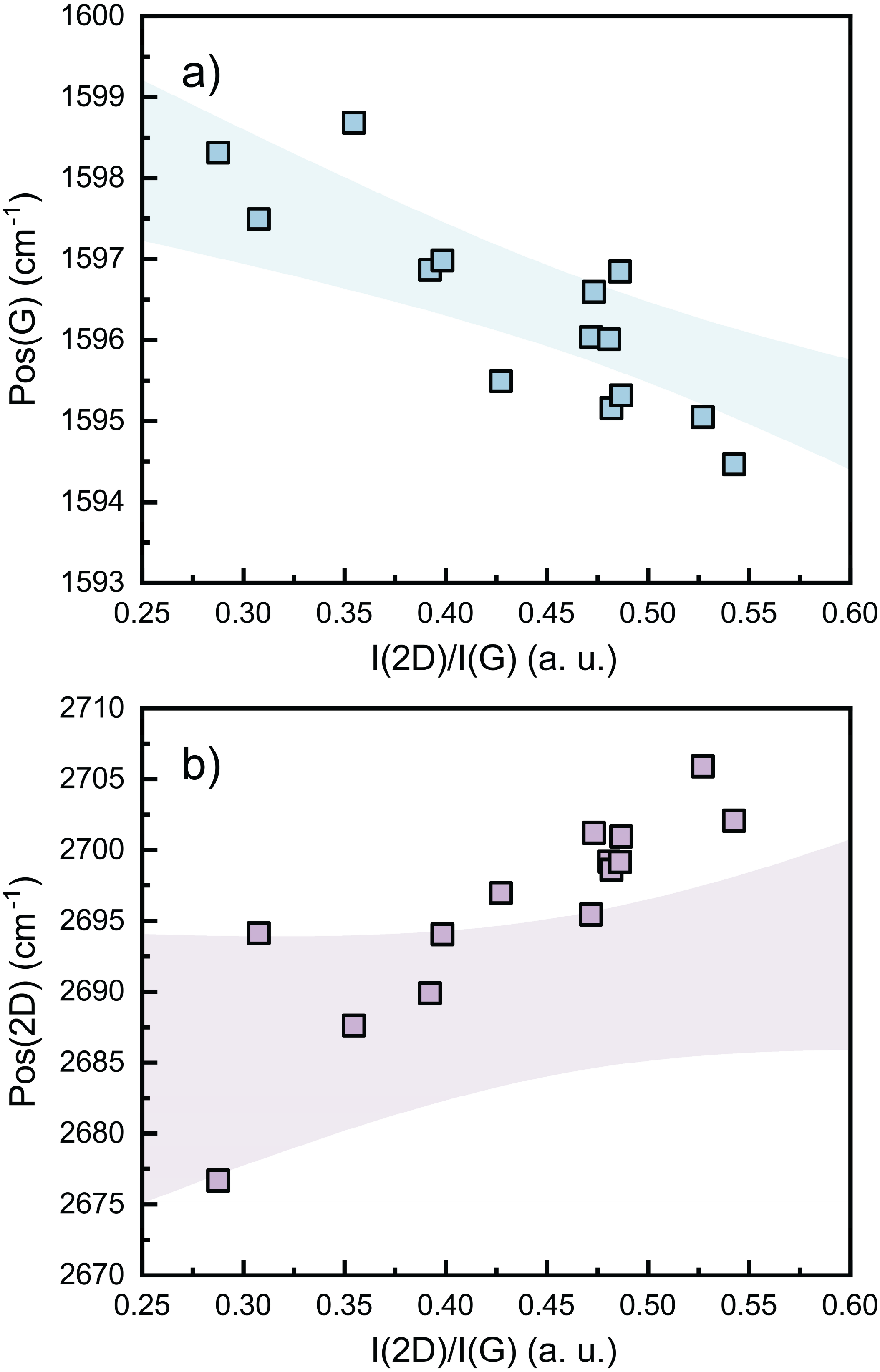

Figure S10. Pos(G) vs. I(2D)/I(G) (a) and Pos(2D) vs. I(2D)/I(G) (b). Shaded area corresponds to a 95% confidence bound.

References

33. Hwang, J.-s.; Lin, Y.-H.; Hwang, J.-Y.; Chang, R.; Chattopadhyay, S.; Chen, C.-J.; Chen, P.; Chiang, H.-P.; Tsai, T.-r.; Chen, L.C.; et al. Imaging layer number and stacking order through formulating Raman fingerprints obtained from hexagonal single crystals of few layer graphene. *Nanotechnology* **2013**, *24*, 015702.
43. Lee, J.E.; Ahn, G.; Shim, J.; Lee, Y.S.; Ryu, S. Optical separation of mechanical strain from charge doping in graphene. *Nat. Commun.* **2012**, *3*, 1024.
59. Zhao W.; Tan P.H.; Liu J.; Ferrari A.C. Intercalation of Few-Layer Graphite Flakes with FeCl3: Raman Determination of Fermi Level, Layer by Layer Decoupling, and Stability. *J. Am. Chem. Soc.* **2011**, 133, 5941–5946.
60. Szirmai P.; Márkus B.G.; Chacón-Torres J.C.; Eckerlein P.; Edelthalhammer K.; Englert J.M.; Mundloch U.; Hirsch A.; Hauke F.; Náfrádi B.; et al. Characterizing the maximum number of layers in chemically exfoliated graphene. *Sci Rep.* **2019**, 9, Article 19480.
61. Childres, I.; Jauregui, L. A.; Tian, J.; Chen, Y. P. Effect of oxygen plasma etching on graphene studied using Raman spectroscopy and electronic transport measurements. *New J. Phys*. **2011**, 13, Article 025008.
62. Sakavičius, A.; Astromskas, G.; Bukauskas, V.; Kamarauskas, M.; Lukša, A.; Nargelienė, V.; Niaura, G.; Ignatjev, I.; Treideris, M.; Šetkus, A. Long distance distortions in the graphene near the edge of planar metal contacts. *Thin Solid Films* **2020**, 698, Article 137850.
63. Kim, S.; Ryu, S. Thickness-dependent native strain in graphene membranes visualized by Raman spectroscopy. *Carbon* **2016**, 100, 283–290.
64. Armano, A.; Buscarino, G.; Cannas, M.; Gelardi, F. M.; Giannazzo, F.; Schilirò, E.; Agnello, S. Monolayer graphene doping and strain dynamics induced by thermal treatments in controlled atmosphere. *Carbon* **2018**, 127, 270–279.
65. Neumann, C.; Reichardt, S.; Venezuela, P.; Drögeler, M.; Banszerus, L.; Schmitz, M.; Watanabe, K.; Taniguchi, T.; Mauri, F.; Beschoten, B.; Rotkin, S. V.; Stampfer, C. Raman spectroscopy as probe of nanometre-scale strain variations in graphene. *Nat. Commun.* **2015**, 6, Article 8429.
66. Lee, U.; Han, Y.; Lee, S.; Kim, J. S.; Lee, Y. H.; Kim, U. J.; Son, H. Time Evolution Studies on Strain and Doping of Graphene Grown on a Copper Substrate Using Raman Spectroscopy. *ACS Nano* **2020**, 14, 919–926.
67. Wu, J.-B.; Lin, M.-L.; Cong, X.; Liu, H.-N.; Tan, P.-H. Raman spectroscopy of graphene-based materials and its applications in related devices. *Chem. Soc. Rev.* **2018**, 47, 1822–1873.
68. Zeng, Y.; Lo, C.-L.; Zhang, S.; Chen, Z.; Marconnet, A. Dynamically tunable thermal transport in polycrystalline graphene by strain engineering. *Carbon* **2020**, 158, 63–68.
69. Mohiuddin, T. M. G.; Lombardo, A.; Nair, R. R.; Bonetti, A.; Savini, G.; Jalil, R.; Bonini, N.; Basko, D. M.; Galiotis, C.; Marzari, N.; et al. Uniaxial Strain in Graphene by Raman Spectroscopy: G peak splitting, Gruneisen Parameters and Sample Orientation. *Phys. Rev. B.* **2009**, 79, Article 205433.
70. Ni, Z. H.; Yu, T.; Lu, Y. H.; Wang, Y. Y.; Feng, Y. P.; Shen, Z. X. Uniaxial Strain on Graphene: Raman Spectroscopy Study and Band-Gap Opening. *ACS Nano* **2008**, 2, 2301–2305.
71. Chugh, S.; Mehta, R.; Lu, N.; Dios, F. D.; Kim, M. J.; Chen, Z. Comparison of graphene growth on arbitrary non-catalytic substrates using low-temperature PECVD. *Carbon* **2015**, 93, 393–399.
72. Eckmann, A.; Felten, A.; Mishchenko, A.; Britnell, L.; Krupke, R.; Novoselov, K. S.; Casiraghi, C. Probing the Nature of Defects in Graphene by Raman Spectroscopy. *Nano Lett.* **2012**, 12, 3925–3930.
73. Pereira, L. M.; Sanchez Rodrigues, V.; Freires, F. G. M. Use of Partial Least Squares Structural Equation Modeling (PLS-SEM) to Improve Plastic Waste Management. *Appl. Sci.* **2024**, 14, 628.
74. Di Leo, G.; Sardanelli, F. Statistical significance: p value, 0.05 threshold, and applications to radiomics—reasons for a conservative approach. *Eur Radiol Exp*. **2020**, 4, 18.
